# Recovery of terbium by *Lysinibacillus* sp. DW018 isolated from ionic rare earth tailings based on microbial induced calcium carbonate precipitation

**DOI:** 10.3389/fmicb.2024.1416731

**Published:** 2024-06-03

**Authors:** Zijun Bian, Wei Dong, Zhoushen Ning, Yuexin Song, Kaijian Hu

**Affiliations:** ^1^Jiangxi Provincial Key Laboratory of Environmental Pollution Prevention and Control in Mining and Metallurgy, Ganzhou, China; ^2^School of Resources and Environmental Engineering, Jiangxi University of Science and Technology, Ganzhou, China; ^3^School of Life Sciences, Jiangxi University of Science and Technology, Ganzhou, China; ^4^Yichun Lithium New Energy Industry Research Institute, Jiangxi University of Science and Technology, Yichun, China

**Keywords:** *Lysinibacillus*, biomineralization, terbium, MICP, ionic rare earth

## Abstract

Microbial induced calcium carbonate precipitation (MICP) is considered as an environmentally friendly microbial-based technique to remove heavy metals. However, its application in removal and recovery of rare earth from wastewaters remains limited and the process is still less understood. In this study, a urease-producing bacterial strain DW018 was isolated from the ionic rare earth tailings and identified as *Lysinibacillus* based on 16S rRNA gene sequencing. Its ability and possible mechanism to recover terbium was investigated by using X-ray diffraction (XRD), scanning electron microscopy (SEM), energy dispersive spectroscopy (EDS), and fourier transform infrared spectroscopy (FTIR). The results showed that the urease activity of DW018 could meet the biomineralization requirements for the recovery of Tb^3+^ from wastewaters. The recovery rate was as high as 98.28% after 10 min of treatment. The optimal conditions for mineralization and recovery were determined as a bacterial concentration of OD_600_ = 1.0, a temperature range of 35 to 40°C, and a urea concentration of 0.5%. Notably, irrespective of CaCO_3_ precipitation, the strain DW018 was able to utilize MICP to promote the attachment of Tb^3+^ to its cell surface. Initially, Tb^3+^ existed in amorphous form on the bacterial surface; however, upon the addition of a calcium source, Tb^3+^ was encapsulated in calcite with the growth of CaCO_3_ at the late stage of the MICP. The recovery effect of the strain DW018 was related to the amino, hydroxyl, carboxyl, and phosphate groups on the cell surface. Overall, the MICP system is promising for the green and efficient recovery of rare earth ions from wastewaters.

## 1 Introduction

The demand for rare earth elements (REEs) has dramatically increased due to their indispensable role in various sectors such as national defense and energy (Middleton et al., 2024). Conventional mining processes are usually accompanied by the generation of industrial liquid wastes rich in REEs, which are released in large quantities into the environment, especially in rivers (Xie et al., 2022). This not only pollutes the environment, but also causes serious loss of rare earth resources. Moreover, in these polluted waters, the concentration of REEs is usually relatively low (less than 0.1 g/L), with only a very small amount of rare earths being recovered from wastewaters for secondary use every year (Kaegi et al., 2021). Therefore, how to enrich and recover rare earths from these industrial wastewaters and dilution sources is the focus of current research.

Rare earth recovery techniques include chemical precipitation, solvent extraction, and ion exchange (Belfqueh et al., 2024; Roa et al., 2024). However, these hydrometallurgical methods require large amounts of water and chemical reagents, resulting in secondary environmental pollution (Danouche et al., 2024). In contrast, low-cost and environmentally friendly microbial methods such as bioenrichment and biomineralization are more advantageous (Yu et al., 2020; Gavrilescu, 2022). For instance, Maleke et al. used the thermophilic bacterium Thermus scotoductus SA-01 to enrich Eu from a 0.5 mM Eu^3+^ solution, completely removing the Eu^3+^ within 10 h and biomineralizing the Eu to Eu_2_(CO_3_)_3_ (Maleke et al., 2019). Microbial induced calcium carbonate precipitation (MICP) through urease hydrolysis is a commonly used method. Urea in the environment is decomposed into CO_3_^2−^ and NH_4_^+^, which ultimately produces CaCO_3_ precipitation in the presence of Ca^2+^ (Lin et al., 2023). Lu et al. have successfully utilized MICP to trap and sequester La^3+^ in spherulites, increasing the recovery of La^3+^ by more than 50% (Lu et al., 2021). MICP can adjust the pH of the solution environment, deprotonate the functional groups on cell surface, and increase the effective sites for the adsorption of metal ions (Hammes and Verstraete, 2002; Fan et al., 2020). In addition, cellular metabolism reduces the free energy required for precipitation to achieve supersaturation during MICP, and ions are also gradually accumulated on the cell surface, which results in a higher concentration of metal ions locally than as a whole, and more conducive to the formation of precipitation (Hoffmann et al., 2021). Therefore, based on the above advantages, there is feasibility and technical reference for a rare earth ion recovery system with MICP as its core.

However, MICP is affected by a variety of factors in the application process, and the existing MICP systems are mostly used in applications such as soil modification, concrete crack repair, and manufacture of new biological materials (Zúñiga-Barra et al., 2022; Isar et al., 2023). Previous studies have showed that MICP system required rapid generation of as much biological CaCO_3_ as possible, but rarely involved its application in recovery of REEs. Wang et al. screened a strain of *Lysinibacillus* from rare earth tailings and used it to induce rare earth tailings cementation. Which not only reduced the hazards of heavy metals and radioactivity, but also simplified the extraction of rare earth elements from tailings slag (Wang et al., 2023). Therefore, it is necessary to obtain efficient urease-producing bacterial strains, establishing a MICP system for the recovery of rare earth ions. Terbium is used in a wide range of applications such as optically active materials, sensors, and biomedical equipment (Jastrząb et al., 2019; Qi et al., 2019; Yang et al., 2021). Tb is also an important material for products such as NdFeB permanent magnet functional materials, fuel cells and electric vehicles (Swati et al., 2023). However, Tb is one of the scarcest rare earth metals, and over-exploitation has led to multiple limitations on the supply of Tb. The demand and price for Tb is growing despite the scarcity of its resources (Swati et al., 2023). The significance of Tb recovery from wastewaters is increasing.

Thus, Tb^3+^ was selected as the enrichment object in this study, and its recovery from aqueous solution was investigated by using a urease-producing bacterial strain, which was screened from ionic rare earth mines in Gannan, China. The effects of various factors on the enrichment and recovery of Tb^3+^ in the MICP process were discussed to determine optimal MICP system parameters. Finally, the recovery mechanism was revealed by comparing the biosorption process and the recovery process of MICP by XRD, SEM-EDS, and FTIR. This study would provide a basic research foundation for the application of MICP for the green recovery of REEs.

## 2 Materials and methods

### 2.1 Isolation and screening of urease-producing bacterial strain

Soil samples were collected from rare earth mines and processed as follows: 10 g of soil samples were dispersed with glass beads in a triangular flask, and 90 mL of sterile water was added. The mixture was then incubated on a shaker for 10 min. After incubation, 1 mL of the supernatant was transferred into an EP tube, and placed in a water bath at 85°C for 10 min, and then cooled in an ice bath for 15 min. 100 μL of the bacterial solution was aspirated and coated onto a Luria-Bertani (LB) medium solid medium according to the gradient of 10^−1^ ~ 10^−6^. It was then incubated overnight in an incubator at a constant temperature of 37°C. The single colony was picked out and streaked on LB solid medium 3 times to obtain the single colony, which was then numbered and preserved.

The single colony was inoculated onto an LB solid medium for activation and incubated for 24 h. The activated single colony was inoculated onto urease agar medium and incubated at 37°C for 24 h. The urease agar medium without urea was configured and the single colony was inoculated onto a plate as a false-positive control. The products of urease hydrolysis of urea in the hydrolysis equilibrium of NH_4_^+^ and OH^−^ will lead to an increase in the pH of the medium. Bacteria with robust urease-producing properties will change the color of the urea agar medium from yellow to red (Dhami et al., 2017; Cui et al., 2022). If the medium without urea also turns red, it indicates a false positive result.

### 2.2 16S rRNA gene sequencing

The screened strains were cultured when they grew to the logarithmic phase, and then sent to Sangon Biotech (Shanghai) Co., Ltd. for 16S rRNA gene sequencing. The strain’s genomic DNA was extracted and the 16S rRNA gene fragment was amplified using universal primers. Universal primers: forward primer AGAGTTTGAT CCTGGCTCAG (SEQ ID NO: 1), reverse primer GGTTACCTTGT TACGACTT (SEQ ID NO: 2). After the products were sequenced, the sequences measured were imported to NCBI for comparison, and a phylogenetic tree was constructed using MEGA11.

### 2.3 Determination of urease activity of the strain

Urease activity was determined by conductivity assay (Whiffin, 2004). 1 mL of bacterial solution was mixed with 9 mL of 1.5 M urea solution. The change in conductivity of the solution was measured for 5 min at 25°C using a conductivity meter. The value of the average change in conductivity (mS/cm/min) over the 5 min measured was multiplied by the dilution factor (10 times), which reflects the enzyme activity of the bacterial solution (mS/cm/min), representing the ability of the bacterial solution to hydrolyze urea. Calculation formulas are as follows:

(1) $$U_{x}=\frac{11.11\times n\times \left(C-C_{0}\right)}{t\times OD_{600}}\;\left(R^{2}=0.9988\right)$$

(2) $$Std\;U_{x}=-2.0942x^{2}+29.226x-60.868\;\left(R^{2}=0.9722\right)$$

(3) $$U=U_{x}\times \frac{Std\;U_{x}}{Std\;U_{7}}$$

Where *U_x_* is the urease activity obtained from the actual measurement (mM/min/OD_600_); *x* is the pH of the actual measurement; *n* is the number of dilutions; *t* is the time of the measurement; *C_0_* is the conductivity before the time of the measurement *t* (mS/cm); *C* is the conductivity after the time of the measurement *t* (mS/cm); 11.11 is the coefficient of fixation; *Std U_x_* is the standardized urease activity at pH = *x* (mM/min/OD_600_); and *U* is the standardized urease activity (mM/min/OD_600_) corrected at pH = 7.

### 2.4 Viable cell number determination

Drop plate method was used to determine the number of live cells (di Salvo et al., 2022). Three suitable dilutions were selected according to the live cell values. Took a 10 μL-drop volume of the dilutions 10^−5^, 10^−6^, and 10^−7^ and transferred it to the sterilized petri dish containing LB solid medium. After the drops were completely absorbed by the medium, the plates were inverted and incubated at 37°C for 24 h. After colonies formed, the plate was taken out and number of colonies was counted. The number of colonies multiplied by the number of dilutions and then multiplied by 100, that is, the number of viable cells contained in each milliliter of the sample.

### 2.5 Mineralization and adsorption of Tb^3+^ by the strain

Mineralization experiment was proceeded as follows: The strain was cultured to logarithmic stage and the culture was centrifuged at 4000 rpm to remove the supernatant. The cell participation was washed 3 times with sterile water, and finally diluted with sterile saline to obtain the bacteria suspension at OD_600_ = 2.0. The bacteria (OD_600_ = 1.0 after mixed) were mixed with a solution containing 400 μM Tb^3+^, 25 mM CaCl_2_, and 2% urea, while keeping the solution volume at 30 mL (Bhattacharya et al., 2018; Zheng et al., 2022). The cells loaded with Tb^3+^ after MICP were obtained by centrifugation at 10,000 rpm for 2 min after incubation at 37°C.

Adsorption experiment: Equal volume of deionized water was used instead of 25 mM CaCl_2_ and 2% urea in the mineralization experiment, while other factors kept the same.

Each experiment was repeated three times, and hydrochloric acid was dropped into the reacted precipitate to verify whether the bacteria underwent a mineralization reaction. After centrifugation, Tb^3+^ concentration in the supernatant was determined by the fluorescence method (Dong et al., 2022), and calculated as follows:

(4) $$R=\left(1-\frac{C}{C_{0}}\right)\times 100\%$$

Where *R* is the recovery of Tb^3+^, *c_0_* is the concentration of Tb^3+^ in the solution before adsorption (μM), and *c* is the concentration of Tb^3+^ in the solution after adsorption (μM).

### 2.6 Effect of MICP conditions on the recovery of Tb^3+^

Time, temperature, bacteria concentration, addition of calcium source (CaCl_2_) and urea were selected as the effect factors for one-factor MICP experiment to recover Tb^3+^. Due to the action of ureolytic bacteria, pH could be quickly adjusted to around 9, so pH value was not adjusted by adding extra alkaline chemicals and was not taken as an effect factor.

#### 2.6.1 Effect of temperature on MICP

The incubation temperature was set as 5, 15, 25, 35, 45, 55, 65, 75, and 85°C, respectively.

#### 2.6.2 Effect of bacterial concentration on MICP

The bacterial concentration (OD_600_) added to the system was set as 0.25, 0.5, 0.75, 1.0, 1.5, and 2.0, respectively.

#### 2.6.3 Effect of Ca^2+^ concentration on MICP

The concentration of CaCl_2_ added to the system was set as 0, 0.5, 1.0, 5.0, 10, 15, and 25 mM, respectively.

#### 2.6.4 Effect of urea concentration on MICP

The concentration of urea added to the system was set as 0, 0.1, 0.5, 1.0, 1.5, and 2.0%.

Under each of the above-mentioned conditions, the mineralization experiment was carried out, and the Tb^3+^revovery rate was determined.

### 2.7 XRD characterization

The solution after MICP was centrifuged (8,000 rpm, 10 min), the supernatant was discarded and the precipitate was collected. The precipitate was washed with deionized water for three times, pre-cooled at −20°C and − 80°C, respectively, and then dried for 24 h in a vacuum freeze-dryer. The powdered samples were fully ground to sieve 300 mesh using a mortar and pestle, and the composition of the precipitate phases were analyzed by X-ray powder diffractometer (XRD) under Cu Kα rays in the range of 5° to 80° (2θ).

### 2.8 SEM-EDS characterization

After centrifugation of the mineralized solution at 8000 rpm for 10 min, the precipitate was collected and transferred into a 2.5% glutaraldehyde solution for fixation, where it was immersed for 2 to 4 h. The supernatant was discarded by centrifugation and washed three times using a phosphate buffer solution. Sequentially, dehydration was performed once each using 30, 50, 70, 85, and 95% ethanol, and twice using 100% ethanol. Each dehydration time was 15 ~ 20 min (Czerwińska-Główka and Krukiewicz, 2021). The sample was replaced with isoamyl acetate two times, each time for 20 min, and then centrifuged to obtain the precipitate, which was made into powder by vacuum freeze-drying.

The sample powder was adhered to a copper sheet with conductive adhesive and sprayed with gold (Au). SEM was used for observation and picture-taking, and EDS was used to scan the sample surface and analyze the elemental composition.

### 2.9 FTIR characterization

The vacuum freeze-dried and dried 24 h powder samples and dried potassium bromide (KBr) were mixed in a ratio of 1: 100, and thoroughly ground with a mortar and pestle. These were placed in a tablet press mold and pressed into transparent sheets on a hydraulic press. The surface functional groups of the samples were determined in the wave number range of 4,000 cm^−1^ ~ 400 cm^−1^ with a resolution of 4 cm^−1^.

## 3 Results and discussion

### 3.1 Screening of urease-producing strain and its urease activity

Fifty-five strains isolated from rare earth mines were inoculated into urease agar medium, and one strain DW018 with strong urease activity was screened. Deep red color was shown in the plate at the growth time of about 12 h. The single colony of strain DW18 was white, round, and flat with a smooth surface. Under the microscope, the strain DW018 was fusiform, capsule-like, with a smooth surface and a length of about 2 ~ 5 μm.

The urease activity of strain DW018 in the logarithmic phase was 2.55 mM/min/OD_600_ according to the conductivity method, which was about one-twentieth of that of *S. pasteurii* (45.42 mM/min/OD_600_) as a control. Compared to previously reported ureolytic bacterial strains, urease activity in the range of 2 to 6 mM/min/OD_600_ can all be selected (Mengistu et al., 2023). The strain DW018 still met the requirements for induced mineralization.

### 3.2 Identification of strain DW018

**Figure 1:**
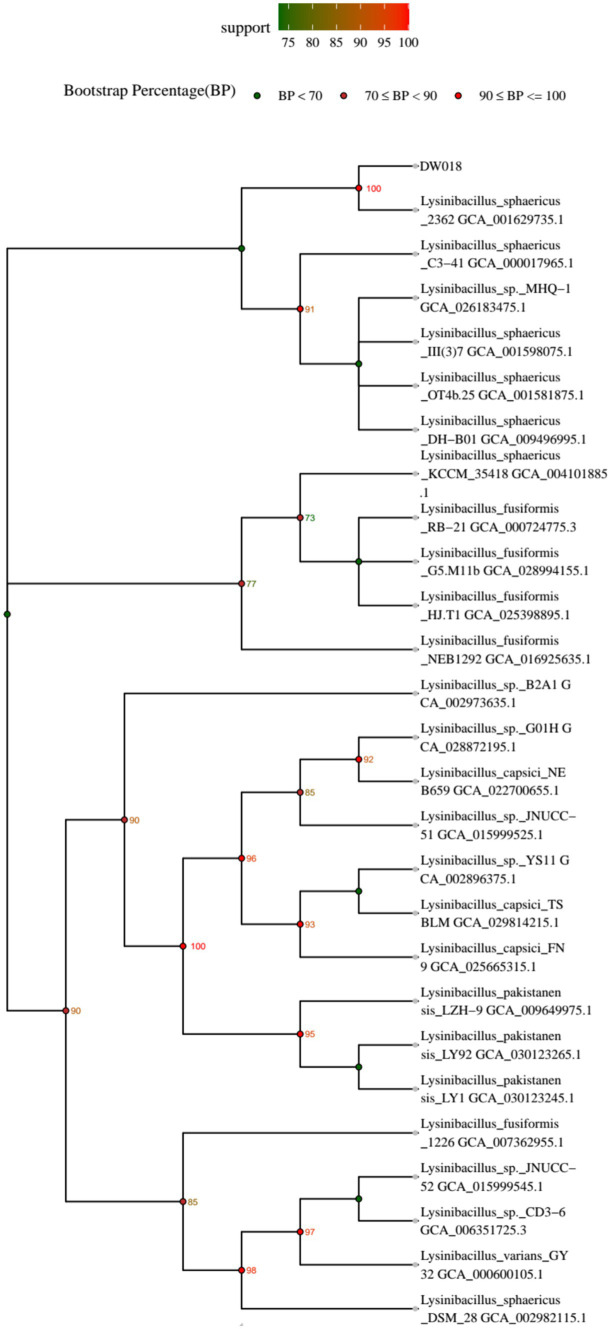
Phylogenetic tree of strain DW018 based on the 16S rRNA gene sequencing.

A phylogenetic tree of the strain was constructed, and the result was shown in Figure 1. It revealed that strain DW018 had the highest similarity with *Lysinibacillus sphaericus* and showed about 90% similarities to other species of *Lysinibacillus*. Based on the above results, strain DW018 was identified as *Lysinibacillus* and named as *Lysinibacillus* sp. DW018.

### 3.3 Mineralization and adsorption of Tb^3+^ by strain DW018

**Figure 2:**
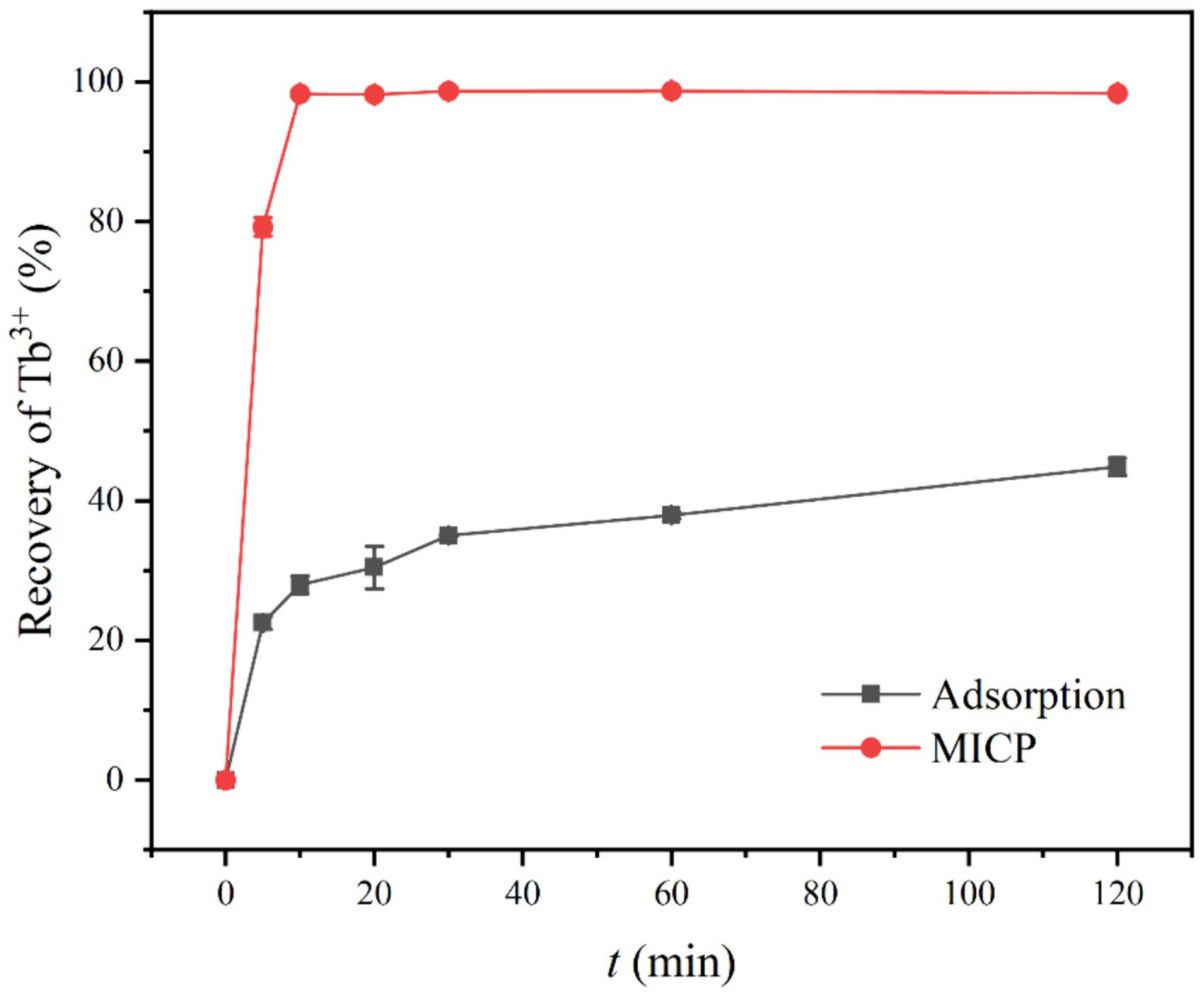
Recovery of Tb^3+^ from aqueous solution. The initial Tb^3+^ concentration of 400 μM, was approximately the concentration of rare earth ions in the wastewaters.

The effects of adsorption and mineralization of strain DW018 on Tb^3+^ in the recovered solution were investigated under the conditions of bacterial solution OD_600_ = 1.0, adsorption time of 2 h, temperature of 37°C, and initial concentration of Tb^3+^ of 400 μM. The results were shown in Figure 2. The recovery rate of mineralization of DW018 rapidly reached 79.22% when *t* = 5 min, while the recovery of adsorption was 22.49%, indicating that MICP can enhance the enrichment of Tb^3+^ in a short time. When *t* = 10 min, the recovery rate by mineralization reached the highest (98.28%) and remained stable, while the recovery by adsorption continued to increase slowly. However, one previous study investigated that complete removal of rare earth ions by biomineralization took around 24 h (Cheng et al., 2018). The results in this study showed that although the adsorption capacity of strain DW018 on Tb^3+^ was not strong, and the promotion effect of MICP on the recovery of Tb^3+^ by DW018 was very significant, increasing the recovery rate of Tb^3+^ by strain DW018 by at least 53.49%.

There are many claims about the mechanism of metal ion enrichment by MICP (Li et al., 2013; Dhami et al., 2017; Lin et al., 2023). It is possible that due to MICP, the cell surface is induced to generate loose and porous CaCO_3_. These CaCO_3_ increased the specific surface area of the cells and promoted the adsorption of Tb^3+^ (Hu et al., 2021; Lu et al., 2021). However, the concentration of CO_3_^2−^ produced by urea decomposition in a short period of time does not reach the solubility product (K_sp_) required for CaCO_3_ precipitation. In addition, CaCO_3_ precipitation could cover adsorption sites, thus crowding out the Tb^3+^ sites (Zhe et al., 2022). Taken together, the recovery of Tb^3+^ seemed to be inhibited. According to the results, the effect of MICP to increase recovery of Tb^3+^ was speedy and long-lasting. Therefore, it is speculated that in the early stage of MICP, urease in bacteria catalyzes the decomposition of urea and produces CO_3_^2−^ and NH_4_^+^, leading to an increase in the pH of the solution environment. The deprotonation of the cell surface groups and the increasing number of active sites continuously attract metal ions to attach to the cell surface (Zeng et al., 2023). The time required for this experiment was short (10 min), the change in pH made by MICP was not significant. In one case study of biomineralization and bioconcentration, Eu^3+^ was not completely removed until the bacterial grew at 10 h, while the pH was maintained around 7 throughout the growth period and only a small amount of Eu_2_(CO_3_)_3_ was produced (Maleke et al., 2019). Similarly, the urease activity of strain DW018 was relatively weak compared with that of *S. pasteurii*, resulting in generation of CaCO_3_ precipitation at a slower rate. Before the mineral completely covered the cell surface, Tb^3+^ was firmly and rapidly adsorbed on the sites and was encapsulated with the cell as the mineral crystals grow.

### 3.4 MICP system optimization

#### 3.4.1 Effect of temperature on Tb^3+^ recovery

**Figure 3:**
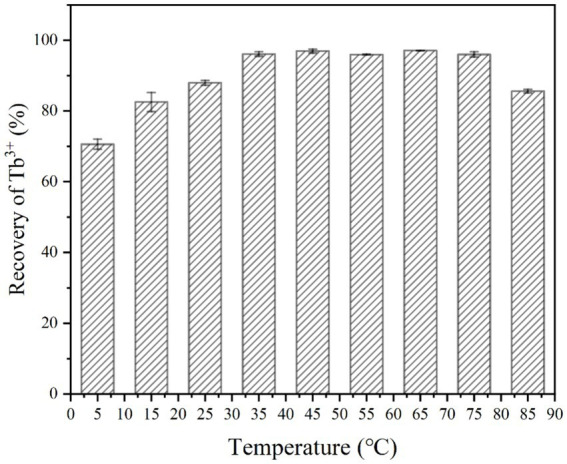
Effect of temperature on Tb^3+^ recovery (OD_600_ of 1.0, time of 10 min, initial Tb^3+^ concentration of 400 μM, CaCl_2_ concentration of 25 mM, and urea concentration of 2.0%).

The experiment evaluated the effect of temperature on Tb^3+^ recovery by strain DW018 after a 10-min mineralization reaction. The results were shown in Figure 3, where the recovery rate appeared an increasing trend as the temperature increased from 5°C to 35°C. When the temperature was low, both bacterial and urease activities were inhibited. Much of the recovery effect on Tb^3+^ at this time was attributed to cell adsorption. When the temperature reached 35°C, the recovery rate of Tb^3+^ reached 96.11%. It was worth noting that strain DW018 exhibited good recovery ability (>96%) under higher temperature conditions (from 35°C to 75°C). Until the temperature reached 85°C, the recovery rate decreased by 10.41%. In conclusion, the optimal temperature range for mineralization recovery of Tb^3+^ was 35°C ~ 75°C.

The temperature that produced significant differences in effect was mainly within 5°C ~ 35°C and 75°C ~ 85°C. The reason for this is mainly that enzyme activity is greatly affected by temperature. Urease activity was weak when the temperature was low, and it was rapidly inactivated when the temperature was high. Suitable temperature range is required by microorganisms for growth. When the temperature is too high, the proteins that make up the various structures and enzymes of microbial cells are inactivated by heat, thus affecting the metabolic processes of bacteria (Ezzine et al., 2024). However, the recovery of Tb^3+^ by strain DW018 did not seem significantly affected by high temperatures. Table 1 showed the number of live bacteria after treating at four temperatures for 10 min. When the temperature reached 45°C, the number of live bacteria decreased almost 200 times of that before heat treatment. When the temperature increased, the number of live bacteria decreased sharply, reaching near-total cell death at 85°C. Combined with the results in Figure 3, it could be seen that the activity of the bacteria did not markedly affect the recovery of Tb^3+^. The cellular structure observed by microscopy remained intact, and the relevant adsorption groups and sites were not destroyed by the short-term high temperature.

**Table 1:** Number of live bacteria after treating strain DW018 at four temperatures for 10 min.

Temperature (°C)	37 (Untreated)	45	65	85
Number of live bacteria (CFU/mL)	8.08 × 10^7^	3.75 × 10^5^	4.63 × 10^4^	0

Urease activity may also be independent of cellular activity because urease is an intracellular enzyme and urease can still function after cell death and lysis (Kim et al., 2018). Table 2 showed the urease activity of strain DW018 at 25°C after pretreatment with four temperatures for 10 min. The results demonstrated that strain DW018 had almost no urease activity after the temperature reached 45°C. In Tables 1, 2, strain DW018 and its urease was not heat-resistant, and even if the cells lysed and died, the urease could not function because of inactivation at high temperatures. However, the results in Table 2 showed the urease activities after the cells were pretreated at high temperature for 10 min, whereas the results in Figure 2 were obtained by directly interacting the cells with Tb^3+^ at high temperature for 10 min. Urease inactivation takes some time, and Feder et al. found that the plant urease jack bean meal urease (JBM urease) was inactivated ≥50% within 5.2 min by 80°C (Feder et al., 2020). Therefore, it could be speculated that, on the one hand, urease had a faster efficiency and rate compared with other relatively efficient C-N hydrolases (Krajewska, 2009). Urease in strain DW018 was not completely inactivated within 10 min and could still catalyze efficiently. On the other hand, the decomposition of urea was accelerated by high temperatures. The two together led to the continuous decomposition of urea to increase the pH, which continuously promoted the attraction of Tb^3+^ to the cellular sites that were not damaged by high temperature. To reduce energy consumption and maintain cells and urease activity, it is advisable to set the incubation temperature within the range of 35 ~ 40°C, consistent with the cultivation conditions of strain DW018.

**Table 2:** Urease activity of strain DW018 at 25°C after pretreatment with four temperatures for 10 min.

Temperature (°C)	25	45	65	85
Urease activity (mM/min/OD_600_)	2.55	0.156	0	0

#### 3.4.2 Effect of bacterial concentration on Tb^3+^ recovery

**Figure 4:**
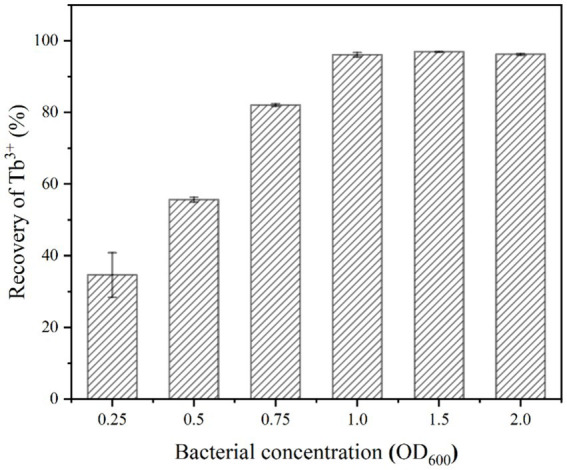
Effect of bacterial concentration on Tb^3+^ recovery (temperature of 37°C, time of 10 min, initial Tb^3+^ concentration of 400 μM, CaCl_2_ concentration of 25 mM, and urea concentration of 2.0%).

The number of effective sites and urease activity in the mineralization system were determined by the bacterial concentration. The effect of bacterial concentration on the recovery of 400 μM Tb^3+^ was shown in Figure 4. It could be seen that when the bacterial concentration (OD_600_) was 0.25 ~ 1.0, the recovery rate of Tb^3+^ appeared an increasing trend up to 96.11%. When the bacterial concentration (OD_600_) continued to increase to 1.5 ~ 2.0, the recovery of Tb^3+^ remained unchanged.

When the concentration of Tb^3+^ was fixed, the above phenomenon could be attributed to two primary factors, including the effective sites and urease activities. Firstly, the high concentration of bacteria had provided enough adsorption sites to adsorb almost all Tb^3+^ to the cell surface (Dong et al., 2022); secondly, the highly active urease rapidly decomposed urea and drove the MICP, which continuously enriched metal ions in solution to the cell surface. Therefore, the bacterial concentration (OD_600_) was optimized at 1.0 based on the amount of rare earths to be recovered and cost considerations.

#### 3.4.3 Effect of Ca^2+^ concentration on Tb^3+^ recovery

**Figure 5:**
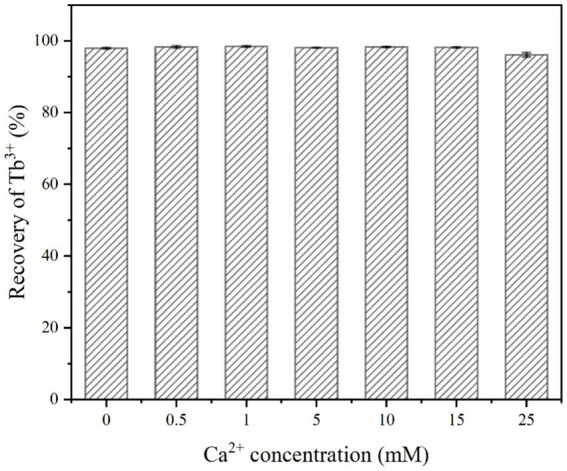
Effect of Ca^2+^ concentration on Tb^3+^ recovery (temperature of 37°C, OD_600_ of 1.0, time of 10 min, initial Tb^3+^ concentration of 400 μM, and urea concentration of 2.0%).

As shown in Figure 5, the recovery of Tb^3+^ by strain DW018 was not affected and the recovery rate remained above 96% regardless of whether Ca^2+^ was added or not. The absence of Ca^2+^ did not interrupt the initial stage of MICP, and urease still decomposed urea to create an alkaline environment and promote Tb^3+^ recovery. On the contrary, high concentrations of Ca^2+^ could affect the osmotic pressure of the cell and interfere with changes in electrical signals on both sides of the membrane, while also possibly inhibit urease activity (Zhao et al., 2019).

During urea hydrolysis, CaCO_3_ is continuously mineralized and deposited around the cell wall due to the presence of Ca^2+^ (Fan et al., 2020). However, it took at least 200 mM Ca^2+^ to significantly reduce bacterial urease activity (Yi et al., 2021). And very little calcium carbonate was generated in a short period of time, which did not lead to a decrease in the permeability of the cell membrane and did not affect the catalytic rate of urease. In addition, the cell surface possessed sufficient sites for Tb^3+^ and Ca^2+^ adsorption. Despite the low concentration of Tb^3+^ in the system (400 μM), the relatively high concentration of Ca^2+^ (25 mM) did not significantly compete with strain DW018 for the recovery of Tb^3+^.

#### 3.4.4 Effect of urea concentration on Tb^3+^ recovery

**Figure 6:**
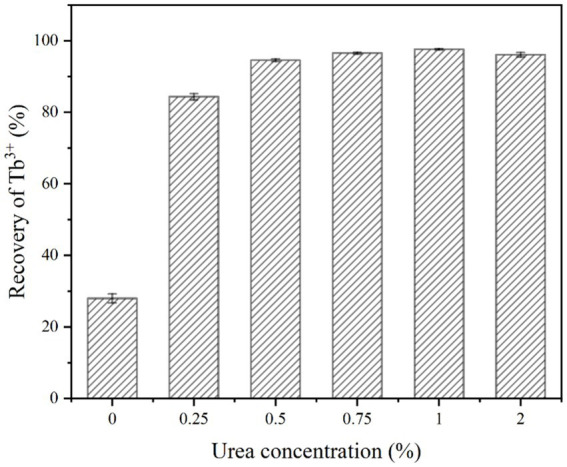
Effect of urea concentration on Tb^3+^ recovery (temperature of 37°C, OD_600_ of 1.0, time of 10 min, initial Tb^3+^ concentration of 400 μM and Ca^2+^ concentration of 0 mM).

The effect of urea concentration on the recovery of Tb^3+^ by strain DW018 in the MICP system without calcium was shown in Figure 6. It could be seen that when the urea concentration was 0.25%, the recovery rate of Tb^3+^ was increased by 56.4% compared to no urea. It indicated that a small amount of urea could induce MICP to promote the rapid recovery of Tb^3+^. When the urea concentration was 0.5%, the recovery rate of Tb^3+^ reached 94.55%. However, further increase in urea concentration did not substantially alter the recovery rate of Tb^3+^ when the urea concentration continued to increase.

Adequate CO_3_^2−^ and OH^−^ are necessary precipitation conditions for biomineralization. CO_2_ in the air dissolves very slowly and urea is relatively stable under natural conditions. Once urease-producing bacteria are used, the rate of urea hydrolysis is increased by 10^4^ times (Yi et al., 2021). Therefore, urea, as a necessary additive to MICP, played an important role in promoting Tb^3+^ recovery by MICP. When the urea concentration was increased from 0 to 0.25%, MICP were triggered and began to drive Tb enrichment onto the cells. The concentration of bacteria (OD_600_ = 1.0) determined the amount of urease that catalyzed a limited amount of urea decomposition. Continuing to elevate the urea concentration did not accelerate the rate of MICP. Instead, excessively high urea concentration could be toxic to the cells and inhibit microbial activity (Fan et al., 2020). In addition, the expression of urease genes is also controlled by urea levels. The results of the study showed that urea is an inducer of urease gene expression, and the addition of 0.4% urea leads to approximately a twofold increase in urease production (Changrong et al., 2020). Because of the fast reaction rate and high conversion efficiency of urease, even if low levels of urea could satisfy the induction requirements of MICP (Zeng et al., 2023). In order to minimize the waste of nitrogen sources and the pollution of ammonia-nitrogen, the optimal urea concentration is 0.5%.

### 3.5 Mechanism of recovery

#### 3.5.1 XRD analysis

**Figure 7:**
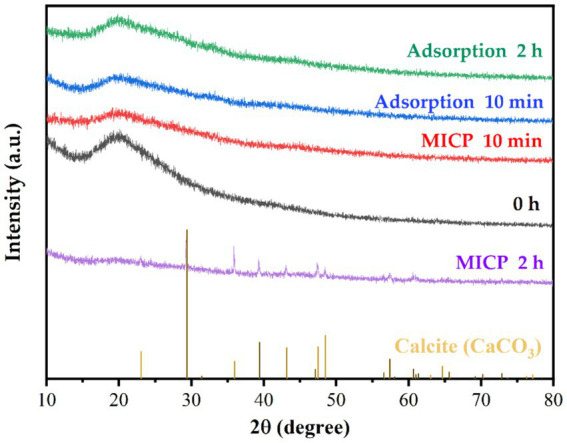
XRD patterns of strain DW018 before and after adsorption and mineralization of Tb^3+^.

The XRD results of strain DW018 before and after adsorption/mineralization of Tb^3+^ were shown in Figure 7. CaCO_3_ was formed gradually with increasing contact time (Zheng et al., 2022). After 2 h of MICP action, characteristic peaks appeared at 2θ = 23.11°, 29.41°, 31.51°, 36.06°, 39.47°, 43.14°, 47.17°, 47.52°, 48.57°, 56.52°, 57.41°, 60.74°, and 64.67° on the XRD spectrum, which were similar to calcite (PDF#99–0022), and is in consistence with the results achieved by other studies (Mengistu et al., 2023). The sharp diffraction peaks in the figure also indicated that the precipitated CaCO_3_ exhibited good crystallinity. Other than that, no diffraction peaks of other materials were found on the XRD pattern, indicating that Tb^3+^ did not form minerals or amorphous compounds before and after cellular adsorption, nor in the early stages of the MICP.

#### 3.5.2 SEM-EDS analysis

**Figure 8:**
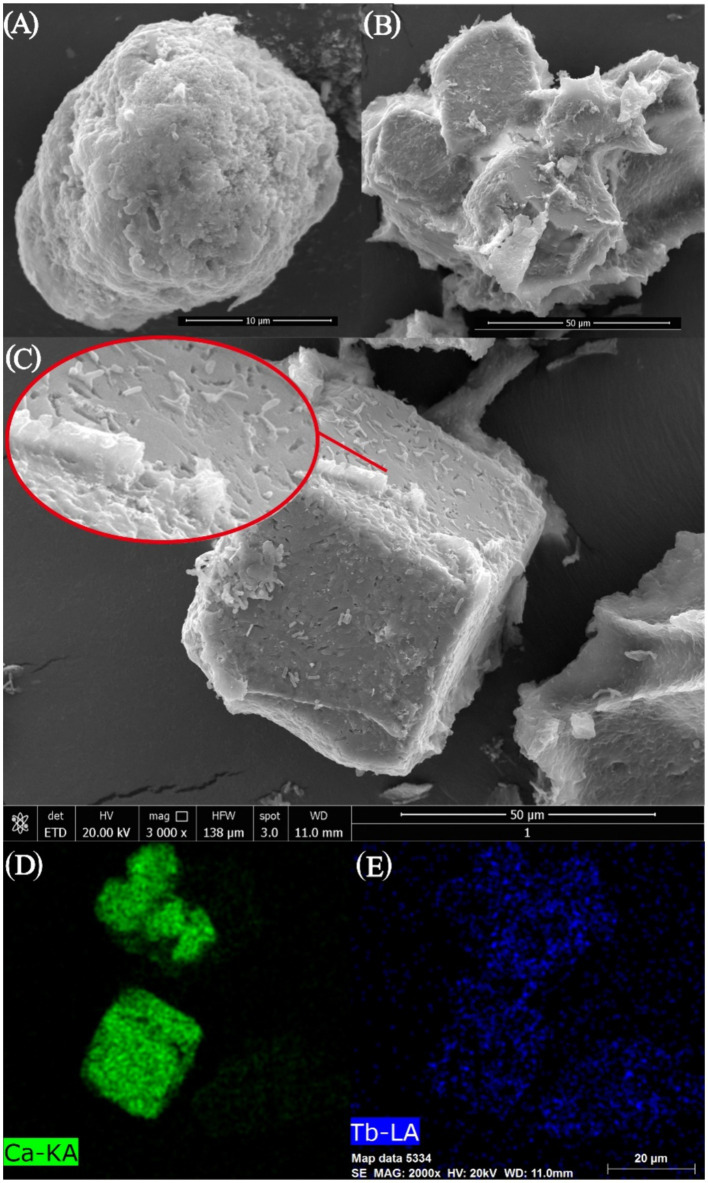
SEM images **(A–C)** and EDS area scan spectrum **(D,E)** of strain DW018 after mineralization when the time is 2 h.

As can be seen from the photographs of CaCO_3_ crystals induced by strain DW018 (Figure 8), square minerals with large diameters and spherical minerals with small diameters would have been produced by strain DW018 after 2 h of MICP. It indicated that the minerals induced by DW018 were mainly calcite, which was consistent with the XRD results. There was no effect of Ca^2+^ concentration on Tb^3+^ adsorption, but the presence of Ca^2+^ induced the production of calcium carbonate crystals. As shown by the photos and the energy spectrum surface scans of calcium carbonate crystals induced by strain DW018 (Figures 8D,E), the surface of calcite was covered or inlaid with a large number of bacteria, providing evidence for the mineralization of urease bacteria (Min et al., 2022). Furthermore, the location of the attached Tb was also on these bacteria. The results suggested that the adsorption of bacteria dominated the process of MICP-promoted recovery of Tb^3+^ (Disi et al., 2022). Previous studies have shown that rare earth ions rarely precipitate as carbonates (Lu et al., 2021; Zhang et al., 2023). Tb cannot be precipitated to form its own insoluble carbonate minerals by the same route, but it can be co-precipitated into a CaCO_3_ crystal lattice by enriching on bacteria.

**Figure 9:**
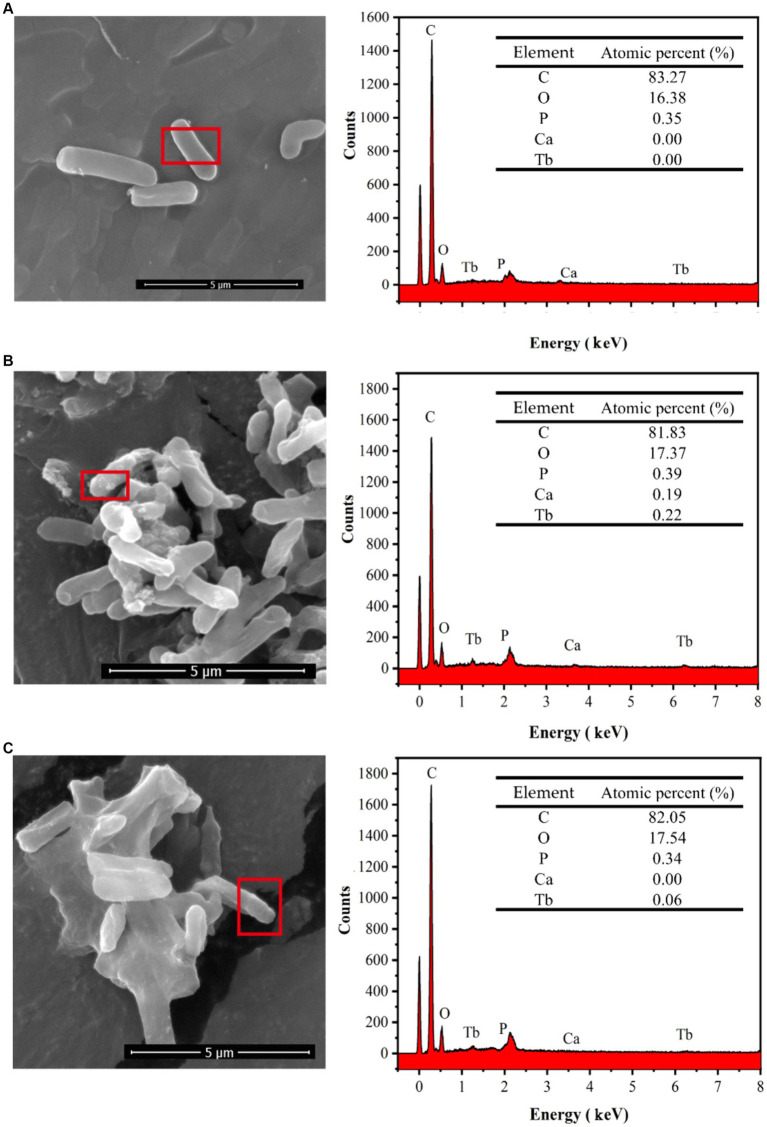
SEM images and EDS spectra of strain DW018 before and after mineralization and adsorption of Tb^3+^. P peak covered by Au. **(A)** Cell, *t* = 0 min; **(B)** MICP, *t* = 10 min; **(C)** Adsorption, *t* = 10 min.

The reaction precipitates at one stage (*t* = 10 min) of strain DW018 mineralization and adsorption of Tb^3+^ were observed by SEM, and the elemental signals on the cell surface were analyzed by EDS, as shown in Figure 9. The cells exhibited a cylindrical shape with a relatively smooth surface before interaction with Tb^3+^ (Figure 9A). After 10 min of MICP interaction (Figure 9B), bright irregular attachments appeared on the cell surface, and the ratio of Ca and Tb elements increased, indicating that Tb^3+^ began to be adsorbed while inducing the production of CaCO_3_. Whereas, after 10 min of cell adsorption (Figure 9C), irregular attachments were present on the cell surface, and no minerals formed either. Gower and Odom first discovered and proposed amorphous calcium carbonate (ACC) as a precursor to CaCO_3_ crystallization with liquid-like properties (Gower and Odom, 2000). Similar ACC was found in cyanobacteria (Mehta et al., 2023). However, ACC is generally found in small amounts in solution, is difficult to isolate and enrich, and is usually present in a mixture with other calcium phases. SEM observations were conducted on freeze-dried solid particulate matter, and it was not possible to determine the presence of similar mobility (Mehta et al., 2023). The materials were presumed to be amorphous mixed Ca-Tb phases.

After biosorption and biomineralization, amorphous Ca-Tb mixed phases were gradually generated on the cell surface, accompanied by an enhancement in the intensity of the Tb elemental signal, which was consistent with the results of existing studies (Fan et al., 2020; Qian et al., 2021; Dong et al., 2022). However, comparing the energy spectra after 10 min of adsorption (Figures 9B,C), the Tb signal intensity was much lower than that after 10 min of MICP, suggesting that MICP had an obvious facilitating role in the recovery of Tb^3+^.

The combined results inferred a MICP process for strain DW018. The nucleation site and CO_3_^2−^ required for MICP was provided by DW018. The breakdown of urea by urease raised the pH in the environment and activated the nucleation sites of the cell (Hammes and Verstraete, 2002). The presence of MICP created favorable conditions for precipitation in the periphery of the cell, such as “supersaturated state” (Hoffmann et al., 2021). And in this way, it continuously induced Tb^3+^ to the cell surface. When Ca^2+^ was present in the solution, the “supersaturated state” formed by MICP co-precipitated with Ca^2+^ to promote rapid attachment of Tb^3+^ to the surface. At this stage, Tb^3+^ was precipitated through the combined action of biosorption and MICP. Free Tb^3+^ was almost completely adsorbed and CaCO_3_ began to form on the cell surface (Lu et al., 2021). In proceeding with continuous MICP, vaterite and calcite began to form and the vast majority of Tb^3+^ was embedded into CaCO_3_ (Lu et al., 2021). Compared to alkaline treatment that required a large amount of chemicals (Maleke et al., 2019), the MICP system in this study only needed 0.5% urea, which was environmentally friendly and low cost. In addition, bacterial properties in MICP system were conducive to create Tb precipitation microenvironments (Hoffmann et al., 2021).

#### 3.5.3 FTIR analysis

**Figure 10:**
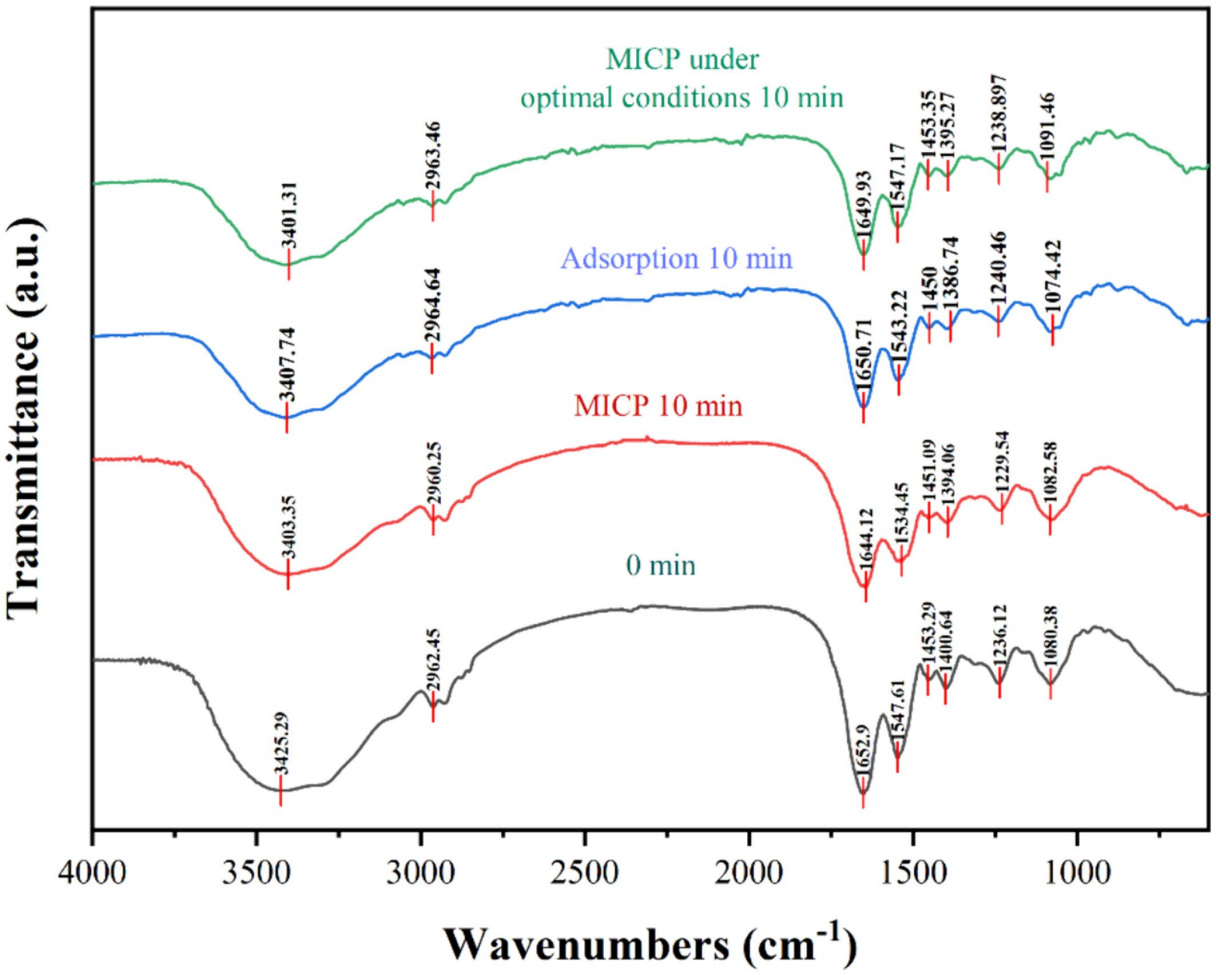
FTIR spectra of strain DW018 before and after mineralization and adsorption of Tb^3+^.

The FTIR spectral characteristics of strain DW018 before and after mineralization and adsorption were shown in Figure 10. The shapes of the peaks of the plots after mineralization and adsorption were the same, compared with that before treatment, suggesting that Tb^3+^ was less abundant and the molecular morphology was unchanged. In addition, the structure of the cell was almost unchanged during the relatively short time of contact with Tb^3+^.

Three spectral bands (1,200 ~ 900 cm^−1^, 1800 ~ 1,500 cm^−1^, and 3,000 ~ 2,800 cm^−1^) were compared separately to analyse the vibrational characteristics of the four groups of precipitates, representing the cells to Tb^3+^ mineralization, mineralization under optimal conditions, adsorption, and the cells before adsorption of Tb^3+^, respectively (İlkay and Zübeyde, 2015; Wang et al., 2017). A broadband vibrational peak shift related to N-H and O-H present near 3,413 cm^−1^ from 3425.29 cm^−1^ → 3403.35, 3407.74, and 3401.31 cm^−1^; the fatty acid in the range of 2,962 ~ 2,919 cm^−1^\u00B0C-H stretching vibration peaks were shifted, from 2962.45 cm^−1^ → 2960.25, 2964.64, 2963.46 cm^−1^; two protein amide bands in the range of 1,653 cm^−1^ ~ 1,541 cm^−1^ and the spectral peaks associated with the PO^2−^groups near 1,072 cm^−1^ (1080.38 cm^−1^ → 1082.58, 1074.42, 1091.46 cm^−1^) were shifted to different degrees. It was known that functional groups such as amino, hydroxyl, carboxyl, and phosphate groups, polysaccharides, lipids, and glycoproteins on the cell wall play an important role in both adsorption and mineralization (Maleke et al., 2019; Dong et al., 2022). Bacterial cells have a variety of charged groups on its surface that attract metal cations (Hoffmann et al., 2021; Wang et al., 2022).

Rare earth ions are also essential metals required by some bacteria (especially methylotrophs) and are selectively taken, transported and inserted into enzymes as active centers (Daumann, 2021; Featherston and Cotruvo Jr., 2021). *Bacillus licheniformis* could selectively remove Sm from the solution containing La, Sm, Er, Lu, and Y (Tsuruta, 2007). It was also found that *Bacillus subtilis* was more selective than *Leisingera methylohalidivorans* and *Phaeobacter inhibens* in the adsorption of Yb and Lu (Breuker et al., 2020). Earlier studies by our research group have shown that *Bacillus* has adsorption and selectivity for a wide range of rare earth ions due to its special structure (Dong et al., 2019, 2022). Therefore, because the strain DW018 has the similar properties, it might have higher selectivity in the recycling application of rare earth ions. However, in this study, the MICP is not used to select but accelerate the enrichment for Tb^3+^ by bacterial cells. For selectivity in real wastewaters, more consideration is given to the characteristics of the bacterial strain used.

## 4 Conclusion

In this study, a bacterium *Lysinibacillus* sp. DW018 capable of MICP was isolated and identified. Carbonate precipitation induced by strain DW018 enabled rapid recovery of Tb^3+^ at low concentrations in solution. Compared with cellular adsorption, MICP could show more than 98% recovery within 10 min. The best recovery of 400 μM Tb^3+^ was achieved when the MICP system was at temperatures ranging from 35°C to 40°C, bacterial concentration OD_600_ = 1.0, and urea concentration at 0.5%. The promotion of Tb^3+^ recovery mainly occurs in the early stage of MICP. Tb is initially enriched on the surface of the bacteria, where functional groups, such as amino, hydroxyl, carboxyl, and phosphoric groups, play important roles. If Ca is present in the MICP system, Tb is immobilized inside the CaCO_3_ crystals as the reaction proceeds. Therefore, in this study, the optimized MICP without calcium source is more suitable for the recovery of Tb^3+^. This optimized method would provide several advantages, including easy controllability, environmental friendliness, low cost, and promising prospects for the recovery of REEs from wastewaters.

## Data availability statement

The authors acknowledge that the data presented in this study must be deposited and made publicly available in an acceptable repository, prior to publication. Frontiers cannot accept a manuscript that does not adhere to our open data policies.

## Author contributions

ZB: Conceptualization, Data curation, Methodology, Validation, Writing – original draft, Writing – review & editing. WD: Conceptualization, Investigation, Methodology, Project administration, Writing – original draft, Writing – review & editing. ZN: Data curation, Validation, Writing – review & editing. YS: Data curation, Investigation, Writing – review & editing. KH: Project administration, Supervision, Writing – review & editing.
